# The Interspecific Competition Between Larvae of *Aedes aegypti* and Major African Malaria Vectors in a Semi-Field System in Tanzania

**DOI:** 10.3390/insects16010034

**Published:** 2024-12-31

**Authors:** Sperancia Coelestine Lushasi, Yohana A. Mwalugelo, Johnson K. Swai, Arnold S. Mmbando, Letus L. Muyaga, Nhandi K. Nyolobi, Anitha Mutashobya, Augustino T. Mmbaga, Hamisi J. Kunambi, Simoni Twaha, Mwema Felix Mwema, Dickson W. Lwetoijera

**Affiliations:** 1 Environmental Health and Ecological Sciences Department, Ifakara Health Institute, Morogoro P.O. Box 53, Tanzania; ymwalugelo@ihi.or.tz (Y.A.M.); skyeba@ihi.or.tz (J.K.S.); ammbando@ihi.or.tz (A.S.M.); lmuyaga@ihi.or.tz (L.L.M.); nnyolobi@ihi.or.tz (N.K.N.); amutashobya@ihi.or.tz (A.M.); ammbaga@ihi.or.tz (A.T.M.); hkunambi@ihi.or.tz (H.J.K.); stwaha@ihi.or.tz (S.T.); 2 School of Life Sciences and Bioengineering, The Nelson Mandela African Institution of Sciences and Technology, Arusha P.O. Box 447, Tanzania; 3 Vector Biology Unit, Epidemiology and Public Health Department, Swiss Tropical and Public Health Institute, Kreuzstrasse 2, Allschwil, 4123 Basel, Switzerland; 4 School of Materials, Energy, Water and Environmental Sciences, The Nelson Mandela African Institution of Science and Technology, Arusha P.O. Box 447, Tanzania; mwema.felix@nm-aist.ac.tz

**Keywords:** interspecific competition, intraspecific competition, *Aedes aegypti*, *An. arabiensis*, *An. gambiae*, *An. funestus*, predation, cannibalism

## Abstract

Interspecific competition between mosquito larvae may affects adult vectorial capacity, potentially reducing disease transmission. It also influences population dynamics, and cannibalistic and predatory behaviors. However, knowledge of interspecific competition between *Ae. aegypti* and *Anopheles* species is limited. The study examined interspecific competition between *Ae. aegypti* larvae and either *An.* *arabiensis*, *An. gambiae*, or *An. funestus* on individual fitness in semi-field settings. The experiments involved density combinations of 100:100, 200:0, and 0:200 (*Ae. aegypti*: *Anopheles*), reared with and without food, in small habitats (8.5 cm height × 15 cm diameter) with 0.5 liters of water and large habitats (15 cm height × 35 cm diameter) with 1 liter of water. The first group received Tetramin^®^ fish food (0.02 g), while the second group was unfed to assess cannibalism and predation. While interspecific competition affected both genera, *Anopheles* species experienced greater effects, with reduced survival and delayed development, compared to *Ae. aegypti*. The mean wing lengths of all species were significantly small in small habitats with a mixed population (*p* < 0.001). The presence of food reduced cannibalism and predation compared to its absence. These interactions have implications for diseases transmission dynamics and can serve as biological indicators to signal the impacts of vector control interventions.

## Simple Summary

*Aedes* and *Anopheles* mosquitoes are major public health threats due to their ability to transmit diseases such as dengue, malaria, yellow fever, Zika, and Chikungunya to humans. Emerging studies are showing the coexistence of these vectors at larval stage particularly in urban areas. Its coexistence may lead to interspecific competition for the limited resources such as food and habitat, impacting their growth rate, development, and survival. This may indirectly and negatively affect the vectorial capacity of adult mosquitoes and reduce or increase the risks of disease transmission thereof. In addition, this phenomenon may influence cannibalistic and predatory behaviors and lead to change in species composition and abundance. This study systematically, examined the effect of interspecific competition between *Aedes aegypti* and major African malaria vectors, such as *Anopheles arabiensis*, *Anopheles gambiae* s.s., or *Anopheles funestus*, on individual fitness in semi-field settings from June to September 2023. The results showed that interspecific competition significantly affected both genera; however, *Anopheles* species were more impacted compared to *Aedes aegypti*.

## 1. Introduction

Mosquitoes, such as *Aedes* and *Anopheles* species, are major public health threats due to their role in transmitting vector-borne disease (VBD), such as malaria, yellow fever, dengue, Zika, and Chikungunya to humans [1]. Globally, around 80% of people are at risk of being affected by at least one VBD that accounts for an estimated 17% of the global burden of infectious diseases and causes about 700,000 mortalities each year [2]. Among vectors that pose significant threats to public health, such as *Aedes* and *Anopheles* species, coexist at the larval stage in urban and suburban areas as documented in previous studies [3,4,5,6,7]. This aligns with WHO malaria report, that infrastructure development can affect the distribution and quality of breeding sites, making mosquitoes adapt to changing environments and become capable of surviving outside their natural aquatic habitats [8]. The preferred breeding habitats for *An. gambiae* and *An. arabiensis* species include small and temporary clean water such as puddles, hoof prints, tire tracks, and rain pools [9] and large, vegetated semi-permanent and permanent aquatic habitats such as swamps, ponds, and river streams for *An. funestus* [10,11]. On the other hand, *Ae. aegypti* prefers man-made or natural habitats such as small containers, tree holes, pitchers, flower pots, roof gutters, and tires, common in urban environments [12,13]. However, there are certain areas where both *Ae. aegypti* and *Anopheles* species have been documented to coexist in urban and suburban settings [4,5,6]. In Gezira Sudan-Barakati, the co-existence of *Anopheles* and *Aedes* larvae during the dry season was observed in leaking water pipes at 40% and 33.9%, while in irrigation channels were 37% and 26.9%, respectively. In El-Kareiba, co-existence in leaking water was 60.8% for *Anopheles* and 17.5% for *Aedes* [5]. Similar trends were documented in a study conducted in Kinshasa, Democratic Republic of Congo, where *Aedes* species coexisted with *Anopheles* in seven breeding sites [4]. Additionally, another study in Nigeria reported that the most abundant species cohabitated in metal cans and earthenware pots were *Ae. aegypti*, *An. gambiae*, and *An. funestus* [6].

When different mosquito species coexist at the larval stage, interspecific competition for limited resources such as food, space (habitat), and oxygen arise. This competitive pressure influences the mosquito larval growth rate, development, survival [14] and behaviors such as cannibalism and predation [15,16,17], thereby affecting species composition and abundance within ecosystem [18,19]. It has been documented that interspecific competition between mosquito’s larvae may indirectly and negatively influence adult life history traits such as vector competence, body size, fecundity, pathogen susceptibility, longevity, flight capability, and overall vectorial capacity, potentially reducing [18,19,20] or increasing the risks of disease transmission [21]. Interspecific competition has been shown to shape the distribution and composition of *Ae. aegypti* and *Ae. albopictus* populations influencing disease incidence [22]. This shift in species composition is crucial for understanding the dynamics of mosquito-borne disease transmission. A laboratory study on interspecific competition between *Ae. aegypti* and *An. stephensi* species reported delayed larval development of *An. stephensi* and high mortality compared to *Ae. aegypti* [23]. While several studies have focused within the same genus of *Aedes* and *Anopheles* [14,15,20,24,25,26], the knowledge of interspecific competition between *Ae. aegypti* and major African malaria vectors is still limited.

The interaction between biotic and abiotic factors collectively shape the population dynamics of adult mosquitoes [27]. On the other hand, this interaction can alter the effects of competition between different species, potentially reducing competition, leading to the coexistence of species, or changing the advantage of one species over the other [25,28,29,30]. The quantity and quality of food among larvae mosquitoes in the habitats greatly determines their survivorship and growth with an impact on adult traits like longevity, body size, flight capacity, or vector competence [31,32]. Habitat size dependent factor has also been reported to also influence development time, survival, and adults body size between *Ae. aegypti* and *Ae. albopictus* [33,34]. Based on environmental variations, vectorial capacity parameters vary temporally and spatially and a single environmental component might have antagonistic effects on several different vectorial capacity parameters [35,36]. For instance, the optimal temperatures for *Plasmodium falciparum* transmission by malaria vectors range from 25 to 28 °C, with a peak in vector competence at approximately 28 °C, while temperatures below 17 °C and above 35 °C decrease malaria transmission due to a shorter mosquito lifespan and reduced parasite development [37]. The temperature may increase vector competence, lower the extrinsic incubation period, and simultaneously shorten adult lifespan [19,38] but also yield more [39] or less competent vectors for pathogens [40]. In *Ae. aegypti* mosquitoes, vector competence for viruses like dengue, Zika, and Chikungunya peaks between 26 and 29 °C, but declines at temperatures below 16 °C and above 36 °C as mosquito survival and development rates decrease [41].

Previous studies have shown that the larval environment, including competition, density, and nutritional factors can significantly influence mosquito vector competence and susceptibility to infection [42,43]. A study on *Ae. aegypti* documented that nutrient limitations during the larval stage impacts competence for arboviruses as malnourished larvae tend to develop adults with reduced immune function, potentially increasing susceptibility to pathogens such as dengue virus [38,44]. Similarly, larval density stress and competition among *Ae. aegypti* larvae have been linked to increased arbovirus susceptibility due to weaker immune responses in adulthood [44,45]. In *An. gambiae* and *An. stephensi*, high larval density and poor nutrition reduce adult body size and immune response, thereby raising susceptibility to *Plasmodium* infection [46,47,48]. Understanding the consequences of interspecific competition between *Ae. aegypti* and major African malaria vectors is not just ecologically significant but also holds epidemiological importance due to their role in disease transmission [49].

Competitive interactions can drive cannibalistic and predatory behavior which are crucial for understanding mosquito population regulation. These behaviors was observed to drastically reduce population size below its carrying capacity contributing to self-population regulation [50,51]. Cannibalism and predation have been documented in Anopheline malaria vectors between the fourth and first instar of *An. stephensi* [52] and the African vectors *An. gambiae* (s.s.) and *An. arabiensis* [53], a possible implication of population dynamics among these species [15]. Interestingly, these behaviors were observed between *An. gambiae s.s*. and *An. arabiensis* not due to food scarcity but rather limited space suggesting that larval cannibalism in these species is intensified by close contact in small larval environments [15,53]. This implies that intraguild predation between the two species may be prevalent in nature and that it is a facultative process unrelated to food scarcity [16]. In *An. gambiae*, cannibalistic behaviors also increased under food deprivation [54]. Additionally, *Culex tigripes* preferred to prey on *Ae. aegypti* larvae over those of other *Culex* or *An. gambiae* Giles [55]. The understanding of cannibalistic behavior, the factors underlying it, and its consequence on larval fitness is therefore of interest for malaria vectors.

To understand the impacts of interspecific competition between mosquito larvae, this study developed the following question; how do the competitive interactions between larvae of *Ae. aegypti* versus *An. arabiensis*, *Ae. aegypti* versus *An. gambiae*, and *Ae. aegypti* versus *An. funestus* affect individual fitness in semi-field settings? To answer the question, the experiments were set up with intraspecific (single species population as a control) and interspecific (mixed species population) competition in small and large habitats, both with and without food.

## 2. Materials and Methods

### 2.1. Study Area

Experiments were conducted between June and September 2023 in a semi-field system (SFS) at the Mosquito City facility of the Ifakara Health Institute (IHI), located in Kining’ina village (8.11417° S, 36.67484° E), of Kilombero district, southern Tanzania. As described in other studies, the SFS is a large, netted cage enclosure with vegetation and breeding habitats that mimic a natural environment [56]. The temperature and relative humidity were recorded daily using a Tiny tag^®^ data logger placed inside the SFS.

### 2.2. Study Design

The study used a full factorial experimental design to determine the effect of competition (intra and interspecific) and habitat size on mosquito fitness parameters, larval developmental time (the number of days from the introduction of the second stage of the larvae until pupation), survival to adult, as well as wing length as a proxy for adult body size. Additionally, the design was used to determine the effect of food, competition, and habitat size on the rate of cannibalism and predation between test species. The factorial design allows for testing all combinations of factors and their levels to assess their individual and interaction effects on the outcome of interest.

### 2.3. Larval Habitats

Two habitat sizes were created from plastic basins; small (8.5 cm height × 15 cm diameter) with 0.5 L of water and large (15 cm height × 35 cm diameter) with 1 L of water were used. The habitats were monitored daily and replenished as necessary to maintain the same volume. The habitats were covered with nets to prevent emerging adult mosquitoes from escaping.

### 2.4. Experimental Procedures

**Figure 1:**
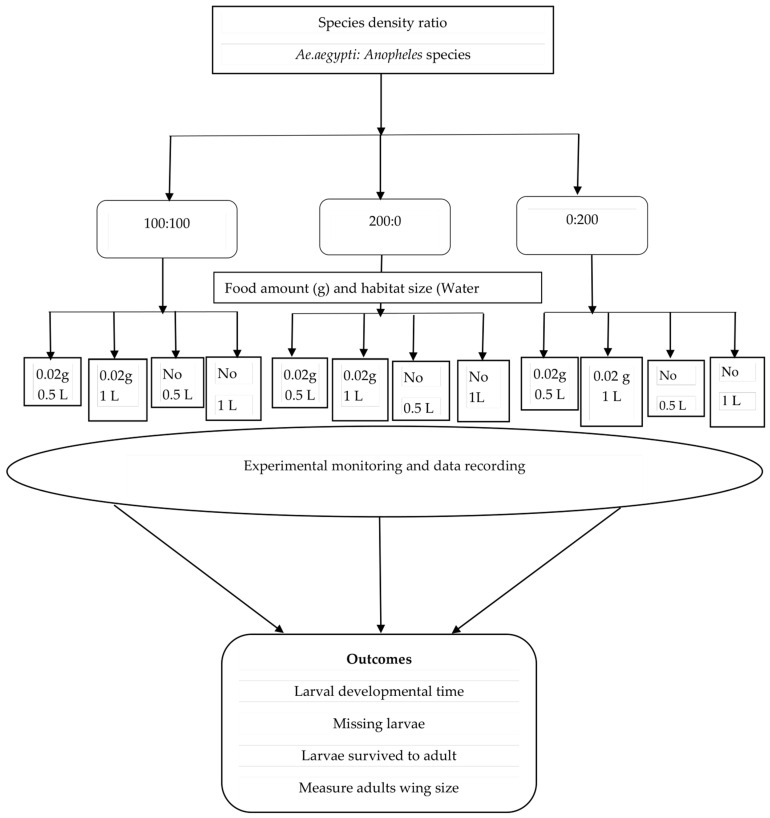
Schematic presentation of the experimental setup and procedures for inter and intraspecific competition between *Aedes aegypti* and either *Anopheles arabiensis*, *Anopheles gambiae*, and *Anopheles funestus* in small and large habitat sizes.

Three mosquito species density combinations; 100:100, 200:0, and 0:200 were set up using *Ae. aegypti* mixed with either (1) *An. arabiensis*, (2) *An. gambiae* s.s., or (3) *An. funestus*. The 100:100 ratios were used to observe interspecific competition between *Ae. aegypti* and *Anopheles* species larvae when reared together in shared habitats. In contrast, the 200:0 and 0:200 ratios represented intraspecific competition that was used as a control, where either *Ae. aegypti* or one of the *Anopheles* species was reared alone. Second-instar larvae for each species and density combination were introduced into both small (0.5 L) and large (1 L) habitats within the semi-field system (SFS) and reared to adulthood. The larvae were assigned either to receive or not to receive food, and this was designed specifically to assess the rate of cannibalistic and predatory behaviors among these mosquitoes in both presence and absence of food. Those selected to receive food were fed Tetramin^®^ fish food (0.02 g) twice per day. The experiments were conducted in two rounds of three replicates each, resulting in a total of six replicates. Each of 12 treatments made a total of 72 larval habitats per species combination and 216 larval habitats for three sets of species combinations (Figure 1). Experimental procedures were identical for all three experiments in both intra and interspecific competition. Additionally, the pilot study confirmed that the food amount and space provided was appropriate for larval growth and survival while still letting the demonstration of the impacts of competition between these two mosquito species based on the metric of an adult’s life history traits at that resource level.

### 2.5. Data Collection

**Figure 2:**
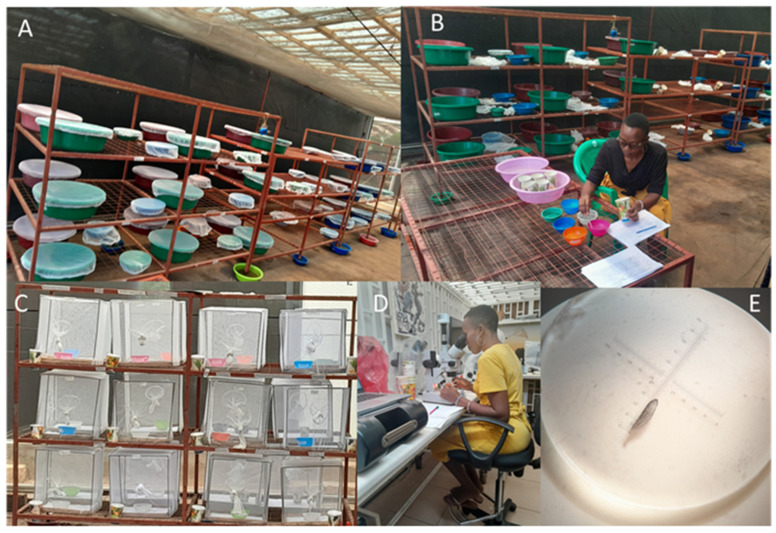
(**A**) Artificial larval habitats consisting of small and large plastic basins used for larval rearing. (**B**) Daily monitoring of larval survival, including counting of alive larvae, dead larvae, missing larvae, alive pupae, and dead pupae. (**C**) Collected pupae were transferred to small plastic bowls with water and placed in net cages for adult emergence. (**D**) Emerged mosquitoes were taken to the laboratory for wing size measurement. (**E**) Wing size observed under a microscope as a proxy for adult body size.

Larval survival was monitored daily by recording the total number alive, dead, and missing (Figure 2). Missing larvae were considered to have been consumed due to cannibalism (in single species population setups, i.e., 200:0 or 0:200) or predation (in interspecific setups, i.e., 100:100), while dead larvae were removed daily and were considered to have died due to natural mortality [57]. After every 24 h, cannibalism was recorded within same species (intraspecific competition) and predation when *Ae. aegypti* were mixed with *Anopheles* species (interspecific competition). The pupae collected daily from each habitat were transferred to small plastic bowls with water (50 mL) and placed in net cages (41 cm height × 35 cm length × 33 cm width) where they were monitored until all emerged as adults or died, a point that marked the end of the experiment. The emerged adults were recorded into their respective species. The number of days between the introduction of larvae into the habitats to pupation were used to estimate developmental time. A subsample of 10 mosquitoes per species for every combination ratio were used for wing length measurement as described in a previous study [44]. A single wing was removed from each female, placed on a glass microscope slide, and measured from the alular notch to the wing tip, excluding the wing fringe. Wing length was measured in millimeters using computer imaging software with a phase contrast microscope. The strong correlation between wing length and dry body weight led to its usage as a body size metric [58,59].

### 2.6. Data Management and Statistical Analysis

The data were analyzed using STATA 18 software (Stata Corp LLC, College Station, TX, USA). The statistical significance was set as a *p* < 0.05. Descriptive statistics, mean and 95% confidence intervals (CI) of developmental time, larvae survival to adulthood, and wing length and missing larvae for each *Ae. aegypti* and *Anopheles* species experiment were calculated.

The percentage larvae survival to adulthood was obtained as the total number of emerged adults divided by the initial number of larvae introduced in the habitat per species multiplied by hundred. Additionally, the number of missing larvae was calculated by first adding up the number of alive larvae, dead larvae, alive pupae, and dead pupae found in the habitat. This total was then subtracted from the number of larvae remaining in the habitat from the previous day to determine how many were missing. To express this as a percentage, the number of missing larvae was divided by the initial number of larvae introduced into the habitat and then multiplied by 100. Larval development time was obtained as the number of days from the start of the experiment (i.e., the date larvae were introduced into the habitats) to pupation time.

Bartlett’s test for homogeneity of variance was conducted and indicated a significant difference in variances among the groups. Therefore, the assumption of homogeneity of variance was violated. Additionally, a Shapiro–Wilk test was used to test the normality of the data indicated that the adult survival and missing larvae data were over dispersed while the development time and wing size data followed a normal distribution. A generalized linear mixed model (GLMM) was used; negative binomial regression was used to examine the fixed effects of competition, habitat, and their interactions on the larvae survived to adulthood, whereby the effect of food was also fitted in the model to account for missing larvae (cannibalism in intraspecific and predation in interspecific). The experimental day was fitted as a random variable in the model to control for daily variability and account for unmeasured factors that could influence the outcome, while competition and habitat size were fitted as fixed variables in the model as well as food. Additionally, for wing size, a generalized linear model following Gaussian (normal distribution) was applied and for development time the mixed effect generalized linear model following Gaussian (normal distribution) was used. GLMM was selected for statistical analysis because it allows for the inclusion of both fixed and random effects making it suitable for analyzing data with repeated measurements over different experimental days.

## 3. Results

The daily recorded temperature and relative humidity inside the semi-field system (SFS) from June to September averaged 27.21 ± 0.05 °C and 74.74 ± 0.16%, respectively.

### 3.1. Larval Developmental Time

The results show that higher levels of competition resulted in prolonged development time for both species. In populations of single species, *An. arabiensis* had a mean (confidence interval) pupation time of 9.7 (9.07, 10.28) days in small and 9.5 (8.80, 10.24) days in large habitats. When reared with *Ae. aegypti*, this increased to 12 (11.35, 12.65) in small and 12.3 (11.79, 12.90) days in large habitats. The time to pupation for *An. arabiensis* was significantly prolonged in mixed populations (RR = 6.11, 95% CI: (2.59, 14.45), *p* < 0.001) compared to single populations (Table 1). For *Ae. aegypti*, the mean time to pupation in single populations was 9.5 (8.84, 10.22) and 9.5 (8.88, 10.12) days in small and large habitats, respectively. In mixed populations with *An. arabiensis*, the time to pupation decreased to 8.5 (7.81, 9.10) and 8.7 (8.14, 9.23) days in small and large habitats, respectively. The time to pupation was reduced in mixed populations of *Ae. aegypti* (RR = 0.54, 95% CI: (0.25, 1.56), *p* = 0.113) compared to single populations with no significant difference. Habitat size had no significant difference on developmental time for both species in single and mixed populations (*p* > 0.05, Table 1).

*An. gambiae* exhibited a mean (CI) pupation time of 7.8 (7.01, 8.51) and 8.17 (7.36, 8.97) days in small and large habitats in populations of single species that increased to 9 (8.17, 9.78) days and 8.9 (8.03, 9.68) days in small and large habitats, respectively, when reared with *Ae. aegypti*. The time to pupation for *An. gambiae* increased in a mixed population (RR = 1.74, 95% CI: (0.74, 4.09), *p* = 0.203) compared to single populations with no significant difference (Table 1). Conversely, *Ae. aegypti* showed a reduced pupation time of 6.7 (6.03, 7.37) and 7.14 (6.38, 7.90) days in small and large habitats, respectively, compared to 8.7 (7.91, 9.43) and 8.7 (7.99, 9.31) days in small and large habitat sizes when reared alone. The pupation time was significantly reduced in mixed populations (RR = 0.30, 95% CI: (0.12, 0.73), *p* = 0.008) compared to single populations (Table 1). Developmental time showed no significant differences between small and large habitats for both *Ae. aegypti* and *An. gambiae* in single and mixed populations (*p* > 0.05, Table 1).

*An. funestus* had a mean (CI) pupation time of 14.4 (13.73, 14.99) and 14.4 (13.71, 14.99) days in both small and large habitats in single populations, as well as 15.5 (14.96, 16) and 15.2 (14.53, 15.82) days in the same habitats when reared with *Ae. aegypti*. However, the time to pupation increased in mixed populations (RR = 1.40, 95% CI: (0.95, 2.07), *p* = 0.088) compared to single populations with no significant differences (Table 1). By contrast, *Ae. aegypti* took longer to pupate in single populations, with mean times of 10.6 (9.97, 11.29) days in small and 11 (10.40, 11.67) days in large habitats, compared to 7 (6.49, 7.59) and 7.7 (7.14, 8.30) days in the same habitats, in mixed populations. The time to pupation was significantly reduced in mixed populations (RR = 0.07, 95% CI: (0.02, 0.28), *p* < 0.001) compared to single populations. No interaction effects between factors were detected for development time (*p* > 0.05), but also the habitat size had no significant impacts across all species (*p* > 0.05, Table 1).

### 3.2. Effects of Competition on Mosquito Larvae Survived to Adults

**Figure 3:**
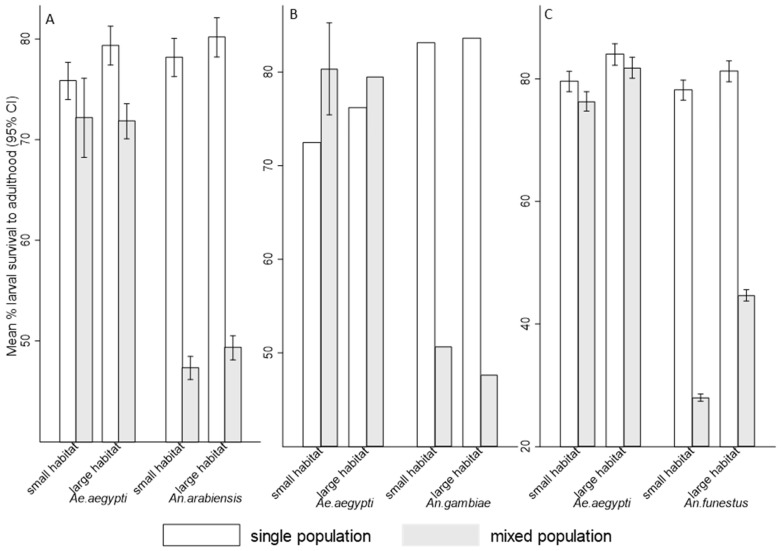
Mean percent (95% CI) of larvae survived to adulthood for (**A**) *Aedes aegypti* and *Anopheles arabiensis*, (**B**) *Aedes aegypti* and *Anopheles gambiae*, and (**C**) *Aedes aegypti* and *Anopheles funestus* exposed in single and mixed population across small and large habitats. Bar graphs without error bars indicate extremely narrow confidence intervals.

The survival rate of *Ae. aegypti*, *An. arabiensis*, *An. gambiae*, and *An. funestus* larvae to adulthood was significantly affected by competition (Table 2). However, *Ae. aegypti* consistently exhibited higher survival rates in interspecific competition compared to *An. arabiensis*, *An. gambiae*, and *An. funestus*, with malaria vectors showing higher larval survival to adults in single species populations than mixed populations (Figure 3A–C). Interspecific competition significantly reduced larval survival to adulthood compared to intraspecific competition for both *Ae. aegypti* (RR = 0.40, 95% CI: (0.30, 0.55), *p* < 0.001) and *An. arabiensis* (RR = 0.23, 95% CI: (0.15, 0.35), *p* < 0.001), *Ae. aegypti* (RR = 0.50, 95% CI: (0.34, 0.74), *p* = 0.001) and *An. gambiae* (RR = 0.43, 95% CI: (0.26, 0.71), *p* = 0.001), as well as *Ae. aegypti* (RR = 0.26, 95% CI: (0.17, 0.39), *p* < 0.001) and *An. funestus* (RR = 0.19, 95% CI: (0.13, 0.28), *p* < 0.001). However, no interaction effects between factors detected by the model to influence the larval survival to adulthood for both genera (*p* > 0.05). The habitat size showed no significant effects in the number of larvae that survived to adulthood for *Ae. aegypti*, *An. arabiensis*, and *An. gambiae* (*p* > 0.05), except *An. funestus* (*p* = 0.024, Table 2).

### 3.3. Adults Body Size via Wing Length (mm)

**Figure 4:**
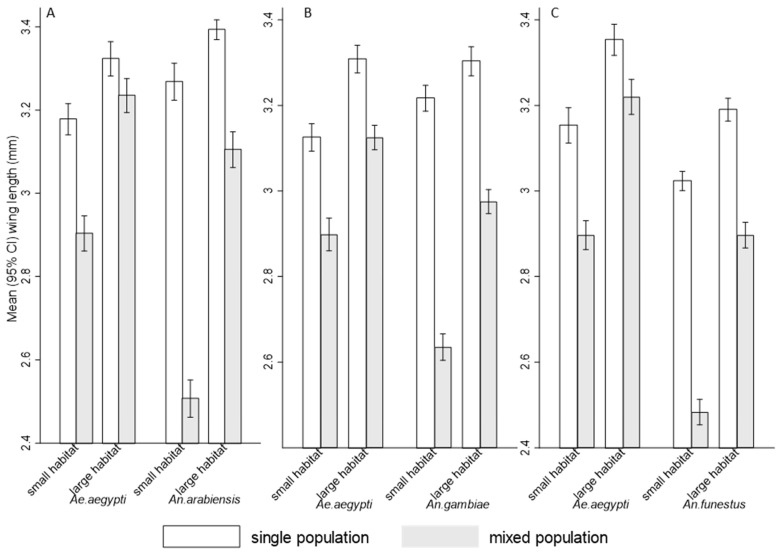
Mean (95% CI) wing length of female (**A**) *Aedes aegypti* with *Anopheles arabiensis*, (**B**) *Aedes aegypti* with *Anopheles gambiae*, and (**C**) *Aedes aegypti* with *Anopheles funestus* in single and mixed population across different habitat size.

The mean wing length of all test species were significantly small for mosquitoes emerging from small habitats compared to those emerging from large habitats in interspecific competition (Figure 4A–C). A mixed species population with small habitats significantly reduced adults’ body size for all tested species compared to large habitats of *Ae. aegypti* (RR = 1.16, 95% CI: (1.09, 1.22), *p* < 0.001) and *An. arabiensis* (RR = 1.13, 95% CI: (1.08, 1.19), *p* < 0.001), *Ae. aegypti* (RR = 0.20, 95% CI: (1.15, 1.26), *p* < 0.001) and *An. gambiae* (RR = 1.09, 95% CI: (1.04, 1.14), *p* < 0.001), as well as *Ae. aegypti* (RR = 1.22, 95% CI: (1.17, 1.29), *p* < 0.001) and *An. funestus* (RR = 1.18, 95% CI: (1.14, 1.22), *p* < 0.001). This highlights the stress of limited space and intensified interspecies competition. In contrast, under intraspecific competition (single species population) mosquitoes from both small and large habitats had significantly larger body size compared to their respective species in interspecific competition with *Ae. aegypti* (RR = 0.76, 95% CI: (0.72, 0.80), *p* < 0.001) and *An. arabiensis* (RR = 0.47, 95% CI: (0.44, 0.50, *p* < 0.001), *Ae. aegypti* (RR = 0.80, 95% CI: (0.76, 0.84), *p* < 0.001) and *An. gambiae* (RR = 0.56, 95% CI: (0.54, 0.58), *p* < 0.001) as well as *Ae. aegypti* (RR = 0.77, 95% CI: (0.73, 0.82), *p* < 0.001) and *An. funestus* (RR = 0.58, 95% CI: (0.56, 0.60), *p* < 0.001), (Figure 4A–C). Adult body size was significantly influenced by the interaction between habitat size and the type of competition (*p* < 0.001, Table 3), with larger body sizes observed when habitat space was increased and competition was intraspecific for both genera.

### 3.4. Cannibalism and Predation Effects

**Figure 5:**
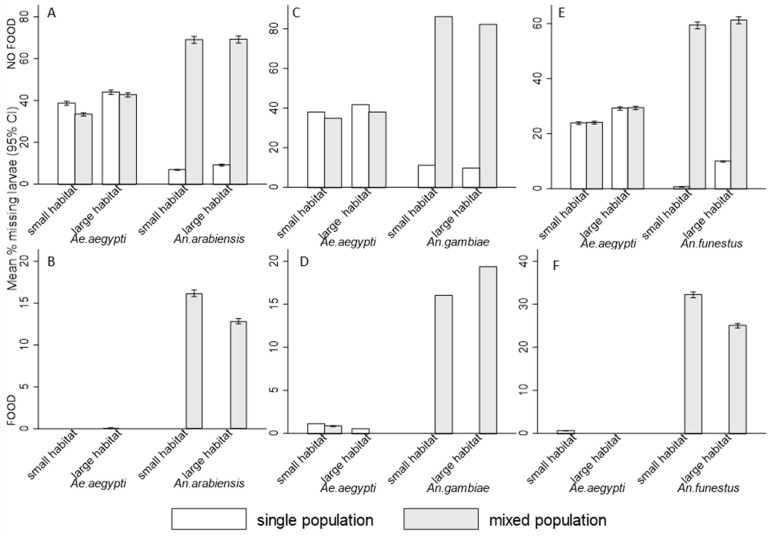
Mean percent (95% CI) of missing larvae in both absence and presence of food for (**A**,**B**) *Aedes aegypti* and *Anopheles arabiensis*, (**C**,**D**) *Aedes aegypti* and *Anopheles gambiae*, and (**E**,**F**) *Aedes aegypti* and *Anopheles funestus* exposed in single and mixed population across small and large habitats. Bar graphs without error bars indicate extremely narrow confidence intervals.

The study observed notable variation in the rate of missing larvae in the presence or absence food; and during competition of *Ae. aegypti* with either *An. arabiensis*, *An. gambiae*, or *An. funestus*. With either presence or absence of food, *An. arabiensis*, *An. gambiae*, and *An. funestus* experienced a higher rate of missing larvae in interspecific competition than *Ae. aegypti* (Figure 5A–F). This indicates that *Anopheles* species encounter greater survival challenges when competing with *Ae. aegypti* than when they are alone. The presence of food decreased the rate of missing larvae in all mosquito species (*p* < 0.001, Table 4, Figure 5A–F). In mixed populations, the risks of being preyed upon or cannibalized were significantly reduced for *Ae. aegypti* and increased for *Anopheles* species; *Ae. aegypti* (RR = 0.54, 95% CI: (0.38, 0.79), *p* = 0.001) and *An. arabiensis* (RR = 8.24, 95% CI: (4.91, 13.83), *p* < 0.001), *Ae. aegypti* (RR = 0.49, 95% CI: (0.36, 0.66), *p* < 0.001) and *An. gambiae* (RR = 6.35, 95% CI: (4.34, 9.29), *p* < 0.001), as well as *Ae. aegypti* (RR = 0.71, 95% CI: (0.49, 1.03), *p* = 0.07) and *An. funestus* (RR = 14.09, 95% CI: (8.55, 23.22), *p* < 0.001), also with significant effects of habitat size on *An. funestus* (*p* = 0.002) (Table 4). The effect of food on cannibalism and predation was observed (*p* < 0.001, Table 4), but no interaction effects between competition, habitat, and food on these outcomes were detected by the model (*p* > 0.05).

## 4. Discussion

This is the first study documenting the effect of competition of cohabiting *Ae. aegypti* and major African malaria vectors at larval stages on mosquito fitness. Overall, the coexistence of *Ae. aegypti* with *An. arabiensis*, *An. gambiae*, or *An. funestus* led to competition, resulting in decreased larval survival, delayed pupation, reduced body size, and an increased rate of missing larvae of *Anopheline* species in competition. When *An. arabiensis*, *An. gambiae*, and *An. funestus* were reared alone, a higher larval survival rate, larger adult body sizes, short pupation time, and reduced rate of missing larvae were observed compared to when they were reared with *Ae. aegypti*. These findings are consistent with previous studies conducted under laboratory and semi-field settings that recorded delayed developmental time, reduced larvae survival rate to adult, and reduced adult body size of cohabiting *Aedes* and *Culex* species [25,60,61,62].

This study documented extended developmental time from larvae to adults as a mosquito fitness cost caused by competition from cohabitation. This could be attributed to intense competition for limited food resources, which reduces food intake per larvae, slowing their growth and delaying pupation. While both species shared the same habitats and resources, this overlap increased closely contact within the same ecological niche that could also be a probable cause of the delayed development. A prior study of *Aedes cantans* under field conditions indicated that frequent contact between larvae could interfere with their feeding, resulting in a prolonged developmental time similar to those caused by food scarcity [63]. In addition, growth-inhibiting cues released by *Ae. aegypti* larvae, which make it grow fast, may prolong the development of *An. arabiensis*, *An. gambiae*, or *An. funestus* [64,65]. Moreover, because some of the food particles given to the larvae tends to settle at the bottom of the larval habitat, *Ae. aegypti* larvae might have an advantage to access these food particles owing to its diving ability compared to coexisting *An. arabiensis*, *An. gambiae*, and *An. funestus*. Additionally, the differences in the mouth brushes of Culicine mosquitoes and the frequency of strokes might result in varying amounts of food intake per unit of time, which in turn might favor shorter developmental compared to *Anopheles* species in the same habitats [66,67]. Subsequently, fast growth rate benefits larval survival by reducing their exposure to vulnerable larvae stages such as cannibalism, predation and environmental factors, i.e., rainfall leading to the flushing of breeding sites or drought periods causing desiccation of larval habitats [15,68].

These findings clearly indicated the effect of food accessibility on larval survival in a mixed population and its emergence to adult mosquitoes. Culicine mosquitoes’ species exhibit more active feeding behavior that could have contributed to their higher resource intake [69]. In a mixed population, *Ae. aegypti* exhibited a higher adult emergence rate than *An. arabiensis*, *An. gambiae*, and *An. funestus*. These results align with previous laboratory studies that examined the effect of interspecific competition within *Aedes* species and between *Ae. aegypti* and *An. stephensi* on survival [23,70]. A separate study has indicated that species capable of sustaining positive population growth tend to hold a competitive advantage over their counterparts [71]. For that case, *Ae. aegypti* outcompeted *An. arabiensis*, *An. gambiae*, and *An. funestus* due to its superior survival rates. The rationale behind this competitive advantage is likely attributed to the enhanced food intake [23,71]. Another study observed a similar trend of *Ae. aegypti* and *Ae. polynesiensis*, whereby *Ae. polynesiensis* showed a competitive advantage over *Ae. aegypti* under field conditions [72]. Similarly, when considering *Ae. albopictus* and *Ae. aegypti*, *Ae. albopictus* maintained a positive population growth over *Ae. aegypti* [73,74].

Interspecific competition in mosquitoes sharing the habitats is recognized as a key factor influencing species distribution and population structure [75,76]. This has been reported in the Southeastern United States, where the reduction in *Ae. aegypti* abundance resulted from its competition with *Ae. albopictus* [77]. The coexistence of *Ae. aegypti* and *Anopheles* species have been observed in natural environments, particularly in the urban and suburban areas of Gezira Sudan, Nigeria, and Kinshasa Congo [4,5,6].

During these experiments, variations in foraging behaviors between test mosquito species were observed. *Ae. aegypti* were observed to predominantly spend more time at the bottom and walls of the larval habitat, whereas *Anopheles* species spent more time at the surface. These behavioral differences could result in differential resource utilization, potentially reducing or avoiding interspecific competition [78]. Similar foraging patterns has been recorded in the coexisting *Ae. albopictus* and *Ae. aegypti*, where *Ae. albopictus* foraged at detritus surfaces, while *Ae. aegypti* occupied the column and bottom of the larval habitat [79]. In addition, *Cx. quinquefasciatus* demonstrated a feeding preference on the lower surface’s microlayer, whereas *An. gambiae* predominantly fed on the upper surface’s microlayer [80,81].

In this study, wing length estimates, revealed that interspecific competition had an effect on body size of all test mosquito species [82,83,84]. Mixed populations in small larval habitats had relatively small body sizes compared to those emerging from large larval habitats. Considering the notable difference in body size of the two mosquito species influenced by habitat size, it is reasonable to infer that limited space serves as a variable in the larval environment, prompting competition that affects the adult mosquito and its associated host seeking, mating, fecundity, and vector competence [34,85,86]

The current study suggest that cannibalism and predation occurred in both *Ae. aegypti* and *Anopheles* species. Cannibalism and predation were established from missing larvae/unrecovered dead larvae. These behaviors between and within *Ae. aegypti* and *Anopheles* species were observed from third day of monitoring at larvae stage three. As *Ae. aegypti* larvae developed faster than *Anopheles* species, they exhibited these behaviors. This is consistent with an earlier study which reported cannibalistic (intraspecific) and predation (interspecific) behavior associated with young (first instar) and old (fourth instar) larvae of three *Anopheles* species [15]. *Anopheles* larvae may be physically less capable of defending themselves against aggressive *Ae. aegypti* making them easier targets for predation. It was observed that *Ae. aegypti* were predating on *Anopheles* probably due to their physical differences, but also to some extent cannibalizing themselves. Because the two species shared the same habitats (small and large habitat) and resources, this overlap increased the chances of predation as both species interacted closely within the same ecological niche. Previous research reported that cannibalism or predation among mosquito larvae may result from the circular currents created by the mouth brushes of older larvae during filtering [87], or through active attacks by conspecifics or heterospecifics and when species are in close proximity [88]. On the other hand, this study suggests that the amount of food given did not affect cannibalism and predation, because few *Anopheles* larvae were missing in the presence of *Ae. aegypti*. This implies that these behaviors are facultative processes and are not dependent on food availability [16].

While the study objectives were achieved, several limitations may have influenced the observed outcomes. The study was conducted in a controlled environment in a semi-field setting designed to mimic realistic conditions. While exposed to fluctuating microclimatic conditions, the environment allowed for control over specific factors, such as predators and varying food availability that could have influenced the outcome. Additionally, laboratory-reared mosquitoes at the second instar stage were used and transferred to the semi-field system, potentially slowing their development as they adapted to the new environmental conditions. Furthermore, the study did not attempt to confirm cannibalism and predation behaviors through methods such as polymerase chain reaction (PCR) analysis of prey DNA, examination of larvae feces, or midguts content analysis. Instead, the study relied on the missing larvae to infer cannibalism and predation, which may have affected the accuracy of these observations.

This study focused on a specific time frame, further studies should investigate the underlying mechanisms driving these competitive interactions, allowing for a more comprehensive understanding of mosquito population dynamics. Further studies should focus on exploring the variables that impact competitive interactions and evaluating the prevalence of such interactions in natural settings. Additionally, other studies should focus on assessing vector competence for dengue and malaria but also other fitness parameters such as fecundity, longevity, host-seeking behavior, and flight capabilities of adult mosquitoes resulting from interspecific competition.

## 5. Conclusions

These findings suggest a competitive advantage in the survival of *Ae. aegypti* over major African malaria vectors. This study has implications for disease transmission dynamics. Environmental changes, such as urbanization, climate change, or human interventions like water management practices in urban areas, can lead to new scenarios where these species do overlap more frequently. Understanding the competitive interactions between *Ae. aegypti* and major African malaria vectors is crucial for predicting changes in the population dynamics of these important disease vectors. For instance, a decline in the *Anopheles* population due to competition can lead to an increase in the *Aedes* population which inadvertently may increase the risks of *Aedes* borne diseases.

## Figures and Tables

**Table 1:** Generalized linear mixed model for the effects of competition, habitat, and their interactions on the developmental time for *Aedes aegypti* and *Anopheles arabiensis*, *Aedes aegypti* and *Anopheles gambiae*, and *Aedes aegypti* and *Anopheles funestus*.

Population	Species	Effects		RR (95% CI)	*p*-Value
*Ae. aegypti* and *An. arabiensis*	*Ae. aegypti*	Competition	Alone	1	
			Mixed	0.54 (0.25, 1.56)	0.113
		Habitat	Small	1	
			Large	1.12 (0.54, 2.30)	0.760
	*An. arabiensis*	Competition	Alone	1	
			Mixed	6.11 (2.59, 14.45)	<0.001
		Habitat	Small	1	
			Large	0.93 (0.44, 1.97)	0.853
*Ae. aegypti* and *An. gambiae*	*Ae. aegypti*	Competition	Alone	1	
			Mixed	0.30 (0.12, 0.73)	0.008
		Habitat	Small	1	
			Large	1.12 (0.49, 2.57)	0.787
	*An. gambiae*	Competition	Alone	1	
			Mixed	1.74 (0.74, 4.09)	0.203
		Habitat	Small	1	
			Large	1.57 (0.65, 3.80)	0.319
*Ae. aegypti* and *An. funestus*	*Ae. aegypti*	Competition	Alone	1	
			Mixed	0.07 (0.02, 0.28)	<0.001
		Habitat	Small	1	
			Large	1.75 (0.81, 3.79)	0.156
	*An. funestus*	Competition	Alone	1	
			Mixed	1.40 (0.95, 2.07)	0.088
		Habitat	Small	1	
			Large	1.00 (0.50, 1.99)	0.977

Note: RR = risk ratio, the interaction row was excluded from the table due to its lack of significant effects but is included in the text to provide a clear interpretation.

**Table 2:** Generalized linear mixed model for the effects of competition, habitats and their interactions on larvae survived to adult for *Aedes aegypti*, *Anopheles arabiensis*, *Anopheles gambiae*, and *Anopheles funestus*.

Population	Species	Effects		RR (95% CI)	*p*-Value
*Ae. aegypti* and *An. arabiensis*	*Ae. aegypti*	Competition	Intraspecific	1	
			Interspecific	0.40 (0.30, 0.55)	<0.001
		Habitat	Small	1	
			Large	0.88 (0.66, 1.16)	0.359
	*An. arabiensis*	Competition	Intraspecific	1	
			Interspecific	0.23 (0.15, 0.35)	<0.001
		Habitat	Small	1	
			Large	0.84 (0.56, 1.29)	0.441
*Ae. aegypti* and *An. gambiae*	*Ae. aegypti*	Competition	Intraspecific	1	
			Interspecific	0.50 (0.34, 0.74)	0.001
		Habitat	Small	1	
			Large	0.89 (0.63, 1.27)	0.55
	*An. gambiae*	Competition	Intraspecific	1	
			Interspecific	0.43 (0.26, 0.71)	0.001
		Habitat	Small	1	
			Large	0.82 (0.52, 1.28)	0.393
*Ae. aegypti* and *An. funestus*	*Ae. aegypti*	Competition	Intraspecific	1	
			Interspecific	0.26 (0.17, 0.39)	<0.001
		Habitat	Small	1	
			Large	0.65 (0.45, 0.94)	0.901
	*An. funestus*	Competition	Intraspecific	1	
			Interspecific	0.19 (0.13, 0.28)	<0.001
		Habitat	Small	1	
			Large	1.02 (0.69, 1.52)	0.024

Note: RR = risk ratio, the interaction row was excluded from the table due to its lack of significant effects but is included in the text to provide a clear interpretation.

**Table 3:** Generalized linear model of the effects of competition, habitat, and their interactions on the adults’ wing length (mm) for *Aedes aegypti* mixed with either *Anopheles arabiensis*, *Anopheles gambiae*, or *Anopheles funestus*.

Population	Species	Effects		RR (95% CI)	*p*-Value
*Ae. aegypti* and *An. arabiensis*	*Ae. aegypti*	Competition	Alone	1	
			Mixed	0.76 (0.72, 0.80)	<0.001
		Habitat	Small	1	
			Large	1.16 (1.09, 1.22)	<0.001
		Competition × Habitat	Mixed × Large		
				1.21 (1.11, 1.31)	<0.001
	*An. arabiensis*	Competition	Alone	1	
			Mixed	0.47 (0.44, 0.50)	<0.001
		Habitat	Small	1	
			Large	1.13 (1.08, 1.19)	<0.001
		Competition × Habitat	Mixed × Large		
				1.61 (1.48, 1.74)	<0.001
*Ae. aegypti* and *An. gambiae*	*Ae. aegypti*	Competition	Alone	1	
			Mixed	0.80 (0.76, 0.84)	<0.001
		Habitat	Small	1	
			Large	0.20 (1.15, 1.26)	<0.001
		Competition × Habitat	Mixed × Large		
				1.04 (0.98, 1.12)	0.196
	*An. gambiae*	Competition	Alone	1	
			Mixed	0.56 (0.54, 0.58)	<0.001
		Habitat	Small	1	
			Large	1.09 (1.04, 1.14)	<0.001
		Competition × Habitat	Mixed × Large		
				0.29 (0.21, 1.37)	<0.001
*Ae. aegypti* and *An. funestus*	*Ae. aegypti*	Competition	Alone	1	
			Mixed	0.77 (0.73, 0.82)	<0.001
		Habitat	Small	1	
			Large	1.22 (1.17, 1.29)	<0.001
		Competition × Habitat	Mixed × Large		
				1.13 (1.05, 1.22)	0.001
	*An. funestus*	Competition	Alone	1	
			Mixed	0.58 (0.56, 0.60)	<0.001
		Habitat	Small	1	
			Large	1.18 (1.14, 1.22)	<0.001
		Competition × Habitat	Mixed × Large		
				0.28 (1.21, 1.35)	<0.001

Note: RR = risk ratio.

**Table 4:** Generalized linear mixed model for the effects of competition, food, and habitats on cannibalistic and predacious behavior for *Aedes aegypti*, *Anopheles arabiensis*, *Anopheles gambiae*, and *Anopheles funestus*.

Population	Species	Effects		RR (95% CI)	*p*-Value
*Ae. aegypti* and *An. arabiensis*	*Ae. aegypti*	Competition	Alone	1	
			Mixed	0.54 (0.38, 0.79)	0.001
		Habitat	Small	1	
			Large	1.15 (0.82, 1.62)	0.423
		Food	No	1	
			Yes	0.001 (0.0001, 0.005)	<0.001
	*An. arabiensis*	Competition	Alone	1	
			Mixed	8.24 (4.91, 13.83)	<0.001
		Habitat	Small	1	
			Large	1.28 (0.79, 2.06)	0.303
		Food	No	1	
			Yes	0.13 (0.07, 0.21)	<0.001
*Ae. aegypti* and *An. gambiae*	*Ae. aegypti*	Competition	Alone	1	
			Mixed	0.49 (0.36, 0.66)	<0.001
		Habitat	Small	1	
			Large	0.86 (0.64, 1.16)	0.326
		Food	No	1	
			Yes	0.02 (0.01, 0.03)	<0.001
	*An. gambiae*	Competition	Alone	1	
			Mixed	6.35 (4.34, 9.29)	<0.001
		Habitat	Small	1	
			Large	1.16 (0.83, 1.63)	0.386
		Food	No	1	
			Yes	0.16 (0.11, 0.24)	<0.001
*Ae. aegypti* and *An. funestus*	*Ae. aegypti*	Competition	Alone	1	
			Mixed	0.71 (0.49, 1.03)	0.07
		Habitat	Small	1	
			Large	0.98 (0.67, 1.45)	0.942
		Food	No	1	
			Yes	0.01 (0.003, 0.013)	<0.001
	*An. funestus*	Competition	Alone	1	
			Mixed	14.09 (8.55, 23.22)	<0.001
		Habitat	Small	1	
			Large	1.99 (1.27, 3.11)	0.002
		Food	No	1	
			Yes	0.26 (0.16, 0.42)	<0.001

## Data Availability

The original data presented in the study are openly available at https://doi.org/10.6084/m9.figshare.27255561.v1. Accessed on 18 October 2024.

## Abbreviations

VBD: Vector-borne diseases, WHO: World Health Organization, IHI: Ifakara Health Institute, SFS: Semi-field system, GLMM: Generalized linear mixed model. CI: Confidence interval, PCR: Polymerase chain reaction, and DNA: Deoxyribonucleic acid.
